# Lower rates of hypoglycaemia in older individuals with type 2 diabetes using insulin degludec versus insulin glargine U100: Results from SWITCH 2

**DOI:** 10.1111/dom.13708

**Published:** 2019-04-15

**Authors:** Simon R. Heller, J. Hans DeVries, Carol Wysham, Charlotte T. Hansen, Melissa V. Hansen, Brian M. Frier

**Affiliations:** ^1^ Academic Unit of Diabetes, Endocrinology and Metabolism University of Sheffield Sheffield UK; ^2^ Academic Medical Center University of Amsterdam Amsterdam the Netherlands; ^3^ School of Medicine University of Washington/Multicare Rockwood Clinic Spokane Washington; ^4^ Novo Nordisk A/S Søborg Denmark; ^5^ The Queen's Medical Research Centre University of Edinburgh Edinburgh UK

**Keywords:** basal insulin, diabetes treatment, elderly, hypoglycaemia, type 2 diabetes

## Abstract

**Aim:**

This study aimed to investigate the safety of insulin degludec (degludec) in relation to age and risk of hypoglycaemia post hoc in individuals with type 2 diabetes (T2D) (SWITCH 2 trial).

**Methods:**

In this crossover study, individuals with T2D who were at risk of hypoglycaemia were randomized to double‐blind treatment with degludec or insulin glargine 100 units/mL (glargine U100) ± oral antidiabetic drugs. After 32 weeks, patients crossed over to the other treatment. Primary endpoint was number of overall severe (positively adjudicated) or glucose‐confirmed (plasma glucose <56 mg/dL; 3.1 mmol/L) symptomatic hypoglycaemia events during the two 16‐week maintenance periods.

**Results:**

For individuals ≤65 (n = 450) and >65 (n = 270) years, baseline median (range) duration of diabetes was 12 (1–40) vs 15 (1–54) years, mean HbA1c was 7.7% vs 7.4% and mean estimated glomerular filtration rate was 87.0 vs 63.7 mL/min/1.73 m^2^, respectively. No significant differences in HbA1c reduction were seen in individuals ≤65 or >65 years. During both maintenance periods, treatment with degludec lowered rates of hypoglycaemia (overall/nocturnal symptomatic) vs those with glargine U100 in individuals ≤65 (31% vs 43%) and >65 (30% vs 41%) years. With degludec and glargine U100, respectively, six vs nine severe hypoglycaemic events occurred in individuals ≤65 years and four vs eight events occurred in those >65 years. Adverse event rates were 3.2 and 3.3 events/patient‐year for individuals ≤65 years and were 3.5 and 4.1 events/patient‐year for individuals >65 years with degludec and glargine U100, respectively.

**Conclusion:**

Treatment with degludec was safe and effective, with a frequency of hypoglycaemia lower than that with glargine U100 in both younger and older individuals (>65 years) with T2D.

## 1 INTRODUCTION

Type 2 diabetes (T2D) is a chronic, progressive disease that frequently necessitates treatment with basal insulin to maintain adequate glycaemic control.^1^, ^2^ In an ageing population with increasing longevity, the global burden of diabetes in individuals 65 years of age or older is projected to increase from 122.8 million in 2017 to 253.4 million in 2045.^3^

Hypoglycaemia, primarily associated with diabetes therapies, particularly insulin, is common in T2D,^4^, ^5^ and increases with age and longer duration of diabetes.^6^ Non‐severe episodes are associated with increased utilization of healthcare services and loss of work time,^7^, ^8^, ^9^ as well as impairment of quality of life; prevention of these episodes is therefore important. Severe hypoglycaemia is of even greater concern, as it has been shown to be associated with increased risk of cardiovascular events and mortality.^10^, ^11^ As with non‐severe events, severe episodes can increase utilization of healthcare resources, with adverse economic consequences.^12^

Delay in intensifying treatment with insulin for many individuals with T2D is common, with fear of hypoglycaemia among patients and healthcare providers an important contributing factor.^13^ The problem of clinical inertia may be magnified in the context of older individuals.^14^ Treatment delay could place older individuals at greater risk of the microvascular and macrovascular complications of diabetes, as they often have less organ reserve and more comorbid conditions than younger individuals.^15^

Basal insulin analogues, now in widespread use, have advantages over human insulin in reducing the risk of hypoglycaemia because of better pharmacokinetic/pharmacodynamic (PK/PD) profiles.^16^ The basal insulin analogue insulin degludec (degludec) has a PK/PD profile with an ultra‐long duration of action,^17^ and these PK/PD properties have been shown to be preserved in elderly individuals.^18^ In type 1 diabetes, degludec has a four‐times lower PD variability than insulin glargine 100 units/mL (glargine U100) (AUC GIR_0‐24h,SS_, coefficient of variation, 20% vs 82%).^19^ The performance of degludec vs glargine U100 has been studied in a large clinical development programme in which degludec was associated with a lower rate of hypoglycaemia, with rate reductions of 17%–86%, compared with glargine U100.^20^ In a dedicated cardiovascular outcomes trial (DEVOTE), a statistically significant 40% lower rate of adjudicated severe hypoglycaemia was observed in individuals using degludec compared with those using glargine U100.^21^

Most randomized trials of insulins include very few older individuals, a population that is heterogeneous with respect to prevalence and severity of comorbidity, frailty and overall health.^22^ Consequently, little information exists concerning the performance of basal insulin analogues in older individuals with diabetes, particularly with respect to hypoglycaemia. A pre‐planned meta‐analysis of seven trials comparing degludec and glargine U100 in older patients (≥65 years) indicated that degludec had a 24% lower rate of overall confirmed hypoglycaemia vs glargine U100, and a 36% lower rate of confirmed nocturnal hypoglycaemia.^23^ Secondary analysis of the DEVOTE trial, which compared degludec with glargine U100, showed that degludec was associated with a lower rate of hypoglycaemia than glargine U100, regardless of age.^24^ A post hoc analysis of data from the SWITCH 2 trial^25^ has been utilized in the present report to explore whether older (>65 years) individuals with T2D responded similarly to younger individuals, with respect to the definitions of hypoglycaemia used in the primary analysis and other safety parameters when comparing degludec with glargine U100.

## 2 METHODS

The detailed design of the SWITCH 2 trial and results of the primary analysis have been published.^19^ Briefly, SWITCH 2 was a randomized, double‐blind, treat‐to‐target, two‐period crossover trial in which adults (≥18 years) with T2D currently undergoing treatment with basal insulin, with or without oral antidiabetic drugs, were randomized 1:1 to receive degludec (Novo Nordisk, Bagsværd, Denmark) once daily and glargine U100 (Sanofi, Paris, France) once daily, in a randomized sequence by period. Participants were also randomized 1:1 within each sequence to a morning dose, between awakening and breakfast, or an evening dose, between the main meal and bedtime. To maintain blinding, both insulins were administered using vial and syringe (100 U/mL, 10 mL vials). Each treatment sequence included a 16‐week titration period and a 16‐week maintenance period. Primary endpoint was the number of overall symptomatic hypoglycaemia (severe or blood glucose‐confirmed [<56 mg/dL {3.1 mmol/L}]) events during the maintenance period.^19^ Other endpoints included the rate of nocturnal symptomatic hypoglycaemia events, severe or blood glucose‐confirmed between 12:01 AM and 05:59 AM (both inclusive) and severe hypoglycaemia, also assessed during the maintenance period. An external, blinded committee positively adjudicated all severe events.

To reflect a broad population of individuals with T2D at risk for hypoglycaemia, inclusion criteria required that individuals must fulfil at least one of the following criteria: at least one severe episode based on American Diabetes Association criteria;^14^ a moderate degree of chronic renal failure (estimated glomerular filtration rate [eGFR] 30–59 mL/min/1.73 m);^2^ reduced awareness of hypoglycaemia; insulin use for more than five years; or experience of a hypoglycaemic event (symptoms and/or blood glucose ≤70 mg/dL [<3.9 mmol/L]) within the previous 12 weeks.

In this post hoc analysis, data from the primary trial^25^ were examined according to age category at baseline; the younger group comprised individuals 65 years of age or younger and the older group comprised individuals above 65 years of age. Statistical analysis was similar to that used in the primary trial. Briefly, a Poisson model with individuals as random effect, with treatment, period, sequence and dosing time as fixed effects, and with logarithm of the observation time as offset was used to estimate the rate ratio for each classification of hypoglycaemia during the maintenance period.^25^ Age group was added to the model as a fixed class variable to facilitate age comparisons pooled across treatments.

## 3 RESULTS

### 3.1 Participants

Among the original cohort, 450 (62.5%) participants were 65 years of age or younger and 270 (37.5%) were above 65 years of age (Table 1). Among younger participants, the distribution according to sex was similar (49.8% female); however, among older participants, there were fewer females than males (42.2% vs 57.8%). At baseline, younger participants tended to be heavier (mean body mass index, 32.8 [5.8] vs 31.2 [5.3] kg/m^2^), to have higher mean HbA1c (7.7% [1.1] vs 7.4% [1.0] {60.3 [12.2] vs 57.9 [11.2] mmol/mol}), to have higher mean fasting plasma glucose (FPG) (7.8 [2.9] vs 7.2 [2.9] mmol/L) and to have a greater prevalence of current smokers (20.0% vs 7.8%). However, younger participants had a shorter median [range] duration of diabetes (12 [1–40] vs 15 [1–54] years) and better renal function (mean eGFR 87.0 [18.9] vs 63.7 [16.8] mL/min/1.73 m^2^) compared with older participants. A much smaller proportion of younger participants had moderate chronic renal failure (10.9% vs 40.7% of younger and older participants, respectively). With respect to any age‐related differences in inclusion criteria relevant to the risk of hypoglycaemia, a larger proportion of older participants had been treated with insulin for more than 5 years (52.6% vs 47.6% of older and younger participants, respectively). However, fewer older participants had experienced at least one severe hypoglycaemia event during the previous year (14.4% vs 17.6% of older and younger participants, respectively). Completion rates were comparable for both younger and older participants, and comparable for both treatments, ranging from 89% to 91%.

**Table 1:** Baseline characteristics, by age group

Characteristic	≤65 years (n = 450)	>65 years (n = 270)
Age (years) median [range]	56.6 [20.9;65.0]	71.5 [65.1;89.2]
Sex (n, %)		
Female	224 (49.8)	114 (42.2)
Male	226 (50.2)	156 (57.8)
Ethnicity (n, %)		
Hispanic or Latino	175 (38.9)	87 (32.2)
Not Hispanic or Latino	275 (61.1)	183 (67.8)
Ethnicity		
White	356 (79.1)	222 (82.2)
Black or African American	65 (14.4)	41 (15.2)
Native Hawaiian or other Pacific islander	1 (0.2)	0 (0)
American Indian or Alaska native	4 (0.9)	3 (1.1)
Asian	19 (4.2)	3 (1.1)
Other	5 (1.1)	1 (0.4)
Body weight (kg)	93.8 (20.3)	88.3 (17.6)
BMI (kg/m^2^)	32.8 (5.8)	31.2 (5.3)
Duration of diabetes (years), median [range]	12 [1–40]	15 [1–54]
Exposure to insulin >5 years (n, %)	214 (47.6)	142 (52.6)
Experience of at least one severe hypoglycaemic event during the previous year^a^ (n, %)	79 (17.6)	39 (14.4)
Impaired awareness of hypoglycaemia b (n, %)	81 (18.0)	48 (17.8)
Experience of at least one hypoglycaemic event^c^ within 12 weeks prior to visit 1 (screening) (n, %)	298 (66.2)	180 (66.7)
eGFR (mL/min/1.73 m^2^)	87.0 (18.9)	63.7 (16.8)
Moderate chronic renal failure^d^ (n, %)	49 (10.9)	110 (40.7)
Smoking status (n, %)		
Never smoked	230 (51.1)	134 (49.6)
Previous smoker	130 (28.9)	115 (42.6)
Current smoker	90 (20.0)	21 (7.8)
HbA1c (%)	7.7 (1.1)	7.4 (1.0)
HbA1c (mmol/mol)	60.3 (12.2)	57.9 (11.2)
FPG (mmol/L)	7.8 (2.9)	7.2 (2.9)
FPG (mg/dL)	140.9 (52.9)	130.6 (51.4)

*Note*. All values are mean (SD) unless otherwise specified.

Abbreviations: BMI, body mass index; CKD‐EPI, chronic kidney disease epidemiology collaboration; eGFR, estimated glomerular filtration rate; FPG, fasting plasma glucose; n, number of subjects; SD, standard deviation.

a Event requiring assistance of another person to actively administer carbohydrate or glucagon, or to take other corrective action. Plasma glucose concentrations may not be available during an event, but neurological recovery following the return of plasma glucose to normal is considered sufficient evidence.

b History of impaired autonomic responses (tremulousness, sweating, palpitations and hunger) during hypoglycaemia.

c Defined by symptoms of hypoglycaemia and/or event with low glucose measurement (≤70 mg/dL [≤3.9 mmol/L]).

d Glomerular filtration rate 30–59 mL/min/1.73 m^2^ per CKD‐EPI by central laboratory analysis.

### 3.2 Comparisons between age groups in the pooled population

**Figure 1:**
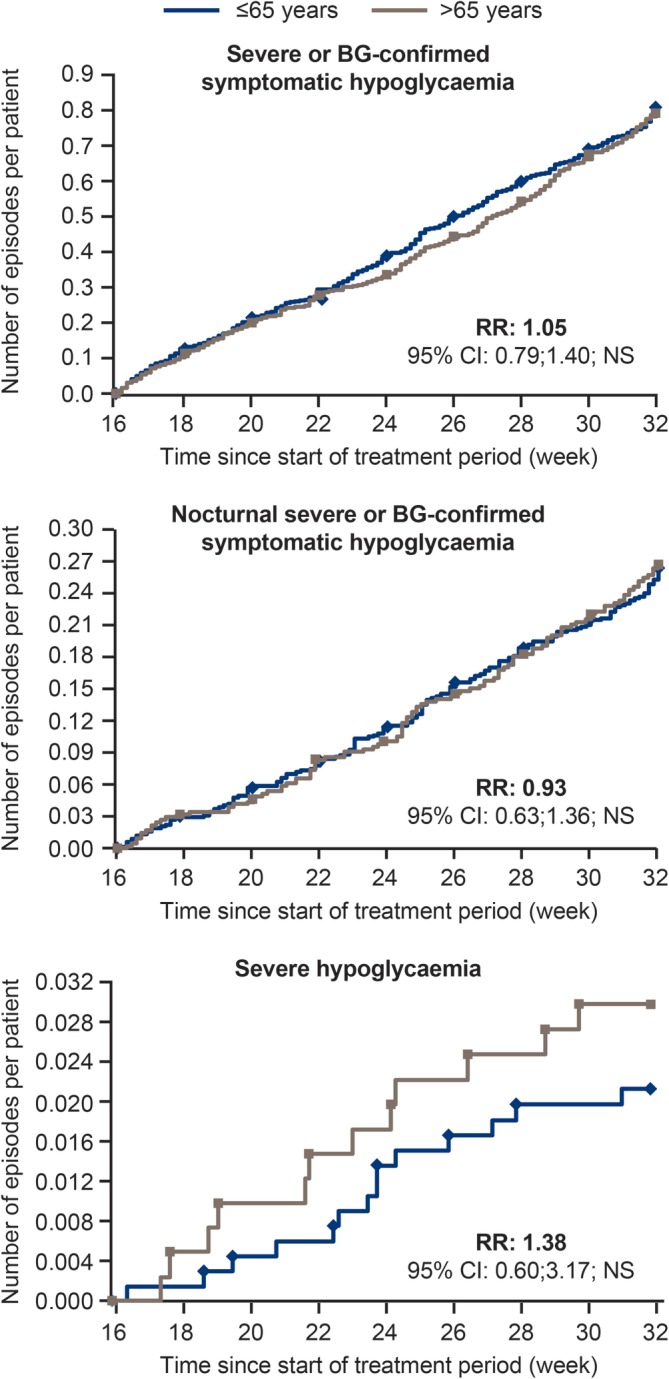
Cumulative number of hypoglycaemic events, for younger (≤65 years) and older (>65 years) individualsAbbreviations: BG, blood glucose; CI, confidence interval; NS, not significant; RR, risk ratio for older: younger individuals. Full analysis set. Values are from the two 16‐week maintenance periods

The cumulative number of hypoglycaemic events, by age group, during the two 16‐week maintenance periods is shown in Figure 1. No statistically significant difference in older vs younger participants was observed in the estimated risk of overall symptomatic hypoglycaemia (relative risk [RR], 1.05 [95% CI, 0.79; 1.40]; *P* = 0.73) or nocturnal symptomatic hypoglycaemia (RR, 0.93 [95% CI, 0.63; 1.36]; *P* = 0.70). Older participants tended to experience more severe hypoglycaemic events, although the difference was not statistically significant (RR, 1.38 [95% CI, 0.60; 3.17]; *P* = 0.45).

Mean basal insulin dose was statistically significantly higher for younger than for older participants throughout the trial (age contrast, older vs younger, −0.14 U/kg [95% CI, −0.21; −0.08]; *P* < 0.001 [period 1] and −0.21 U/kg [95% CI: −0.29; −0.12]; *P* < 0.0001 [period 2]) (Figure S1).

With respect to glycaemic control, both younger and older participants showed reductions from baseline in mean HbA1c; no statistically significant difference was found in change from baseline HbA1c between older and younger participants in treatment period 1 (age contrast, older vs younger, −0.01% [95% CI, −0.15; 0.13]; *P* = 0.91) or in treatment period 2, −0.05% [95% CI: −0.18; 0.08]; *P* = 0.45).

Mean fasting plasma glucose was also reduced from baseline in both age groups. However, the magnitude of decrement was greater for older compared with younger participants during both treatment periods (age contrast, older vs younger, period 1, −0.51 mmol/L [95% CI, −0.87; –0.15]; *P* = 0.0054; period 2, −0.56 mmol/L [95% CI, −1.02; −0.10]; *P* = 0.0168).

### 3.3 Comparisons by treatment within age group

**Figure 2:**
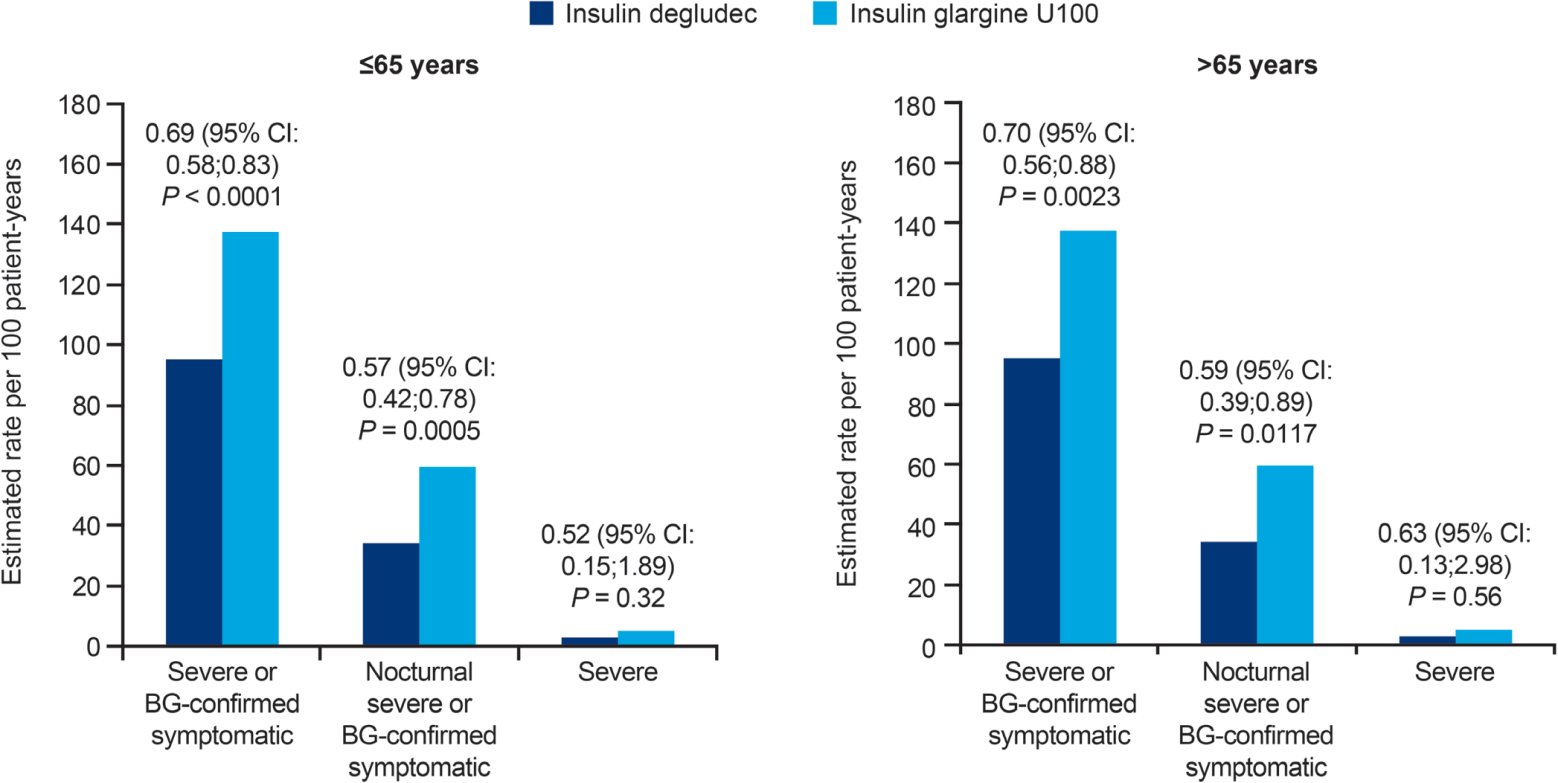
Hypoglycaemic events for younger (≤65 years) and older (>65 years) individuals, by treatment groupAbbreviations: BG, blood glucose; CI, confidence interval.Values are treatment ratios (insulin degludec/insulin glargine U100) for the two 16‐week maintenance periods.*P*‐values derived using a Poisson model with logarithm of exposure time (100 years) as offset; estimates adjusted for treatment period, period sequence and dosing time as fixed effects, and subjects as a random effect

The observed rate of severe or blood glucose (BG)‐confirmed symptomatic hypoglycaemia was lower for degludec compared with glargine U100 in older as well as in younger participants during the maintenance period (younger group, 184 vs 263 events/100 patient‐years of exposure [PYE] for degludec and glargine U100, respectively; older group, 188 vs 269 events/100 PYE for degludec and glargine U100, respectively) (Table 2), with an estimated 31% and 30% lower rates of severe or BG‐confirmed symptomatic hypoglycaemic events with degludec compared with glargine U100, in the younger group (treatment ratio, degludec:glargine U100, 0.69 [95% CI, 0.58; 0.83]; *P* < 0.0001) and in the older group (treatment ratio, degludec:glargine U100, 0.70 [95% CI, 0.56; 0.88]; *P* = 0.0023), respectively (Figure 2). Treatment with degludec was also associated with an estimated 43% and 41% lower rates of nocturnal symptomatic hypoglycaemia than rates with glargine U100, in the younger group (treatment ratio, degludec:glargine U100, 0.57 [95% CI, 0.42; 0.78]; *P* = 0.0005) and in the older group (treatment ratio, degludec:glargine U100, 0.59 [95% CI, 0.39; 0.89]; *P* = 0.0117), respectively (Figure 2). The number of severe events was low in both the younger group (six with degludec and nine with glargine U100) and the older group (four with degludec and eight with glargine U100), and the risk of severe hypoglycaemia was not statistically significantly different for either younger (treatment ratio, degludec:glargine U100, 0.52 [95% CI, 0.15; 1.89]; *P* = 0.32) or older (treatment ratio, degludec:glargine U100, 0.63 [95% CI: 0.13; 2.98]; *P* = 0.56) participants by treatment.

**Table 2:** Summary of hypoglycaemic events for younger (≤65 years) and older (>65 years) individuals, by treatment group

≤65 years (n = 413)	Insulin degludec				Insulin glargine U100				Total			
Type of hypoglycaemia	N	%	E	R	N	%	E	R	N	%	E	R
Severe or BG‐confirmed symptomatic	85	21.4	220	184	118	30.3	310	263	156	37.8	530	223
Nocturnal severe or BG‐confirmed symptomatic	39	9.8	64	53.6	61	15.7	109	92.57	81	19.6	173	72.95
Severe	6	1.5	6	5.02	8	2.1	9	7.64	11	2.7	15	6.33
**>65 years (n = 240)**	**Insulin degludec**				**Insulin glargine U100**				**Total**			
**Type of hypoglycaemia**	**N**	**%**	**E**	**R**	**N**	**%**	**E**	**R**	**N**	**%**	**E**	**R**
Severe or BG‐confirmed symptomatic	57	24.4	133	188	77	33.6	186	269	100	41.7	319	228
Nocturnal severe or BG‐confirmed symptomatic	22	9.4	41	58	30	13.1	66	95.42	44	18.3	107	76.45
Severe	4	1.7	4	5.65	7	3.1	8	11.57	11	4.6	12	8.57

*Note*. Values during the two 16‐week maintenance periods.

Abbreviations: %, percentage of participants; BG, blood glucose; E, number of events; N, number of participants; R, rate (number of events divided by patient‐years of exposure multiplied by 100).

HbA1c decreased with both treatments during the first 16‐week titration period, after which values plateaued (Figure S2). No statistically significant difference was seen between treatments in either age group (degludec–glargine U100, 0.16% [95% CI, −0.02; 0.35]; *P* = 0.07 and − 0.05% [95% CI, −0.26; 0.15]; *P* = 0.60) for younger and older participants, respectively. FPG decreased from baseline during the first 32‐week treatment period for both treatments, after which FPG remained relatively stable across both treatments (Figure S3).

### 3.4 Adverse events

Over the entire trial, in the safety analysis set, 307 of 444 (69.2%) younger patients and 194 of 269 (72.2%) older patients reported adverse events (AEs). The percentage of younger patients reporting AEs was almost identical for degludec and glargine U100 (57.5% and 57.8%, respectively). However, for older patients, a smaller proportion reported AEs with degludec (56.9% vs 66.7% for degludec vs glargine U100, respectively).

Serious AEs by preferred term and system organ class occurring ≥5% in either treatment arm for both age groups are listed in Table S1. These were mostly upper respiratory in nature. There were seven fatal events in total: two in patients treated with degludec (none in the younger group; one associated with sudden cardiovascular death and one caused by stroke in the older group) and five in patients treated with glargine U100 (one secondary to acute myocardial infarction and one caused by sepsis in the younger group; two associated with malignancy and one caused by sepsis in the older group).

## 4 DISCUSSION

Results of the randomized, double‐blind, crossover trial, SWITCH 2, were examined to assess the effect of age on hypoglycaemia risk, comparing degludec with glargine U100. The SWITCH 2 trial was powered to evaluate the superiority of degludec vs glargine U100 with respect to overall symptomatic hypoglycaemia. During the maintenance period, treatment with degludec was associated with statistically significantly lower rates of severe and symptomatic hypoglycaemic events (BG‐confirmed) compared with glargine U100, with a 31% reduction in younger patients (≤65 years) and a 30% reduction in older patients (>65 years). Concerning nocturnal symptomatic hypoglycaemia, the reduction in rates were 43% and 41%, respectively, for these age groups. These treatment differences were comparable between age groups, and this lower rate was similar to the 27% lower rate of overall confirmed hypoglycaemia (estimated rate ratio, degludec:glargine U100, 0.73 [95% CI, 0.56; 0.96]) and the 39% lower rate in nocturnal hypoglycaemia (estimated rate ratio, degludec:glargine U100, 0.61 [95% CI, 0.37; 1.03]) during the maintenance period reported in a pre‐planned meta‐analysis of seven phase IIIa open‐label trials in individuals aged at least 65 years of age, comparing degludec and glargine U100 in T2D.^24^ In contrast to the SWITCH 2 trial, the core trials in the meta‐analysis were powered to detect differences in HbA1c. Nevertheless, these similarly lower rates across trials with heterogeneous patient populations, ranging from insulin‐naïve to basal‐bolus users, with or without an increased risk of hypoglycaemia at baseline, support the overall benefit of treatment with degludec compared with glargine U100.

The number of severe hypoglycaemic events reported in the present analysis were not statistically different by treatment for either age group, probably because the overall number of events was very low. As this was a treat‐to‐target trial, as expected, no treatment differences were observed in change in HbA1c from baseline in either the older or the younger participants. Younger individuals required a higher mean insulin dose (U/kg) compared with older individuals throughout the trial, which may have been related to greater insulin resistance, in line with the tendency of a higher body mass index among younger individuals.

Older individuals with T2D are generally at increased risk of, and more vulnerable to, hypoglycaemia than younger individuals, for several reasons, including impaired renal function,^26^ reduced ability to recognize and respond to hypoglycaemia,^27^ and altered physiological responses to low glucose levels.^28^ Symptoms of hypoglycaemia become less intense and their symptom profile changes with increasing age,^29^ with symptomatic responses manifesting only at BG levels lower than those in younger individuals, leaving less time to recognize and respond to them.^30^, ^31^ Thus, when hypoglycaemia develops in an older individual with T2D, it might not be identified or reported, which may explain the absence of a significantly higher rate of overall hypoglycaemia in older individuals, as was observed in the present study.

Reduced awareness of hypoglycaemia with advancing age may increase the risk that an event progresses in severity and results in more severe events in the older age group. There was a numerically greater rate of severe hypoglycaemia in older individuals compared with younger individuals (RR, 1.38) in the current trial, although the trend was not statistically significant. Severe hypoglycaemia is generally much more common in real‐world populations than in randomized clinical trials.^4^, ^5^, ^32^ In addition, continuous glucose monitoring has demonstrated that many episodes are unrecognized and under‐reported.^33^ Furthermore, it has been estimated that only 5% of self‐reported severe hypoglycaemia events among individuals with diabetes who are underging pharmacological treatment are captured by traditional healthcare utilization‐based surveillance systems, suggesting a substantial underestimate of the true burden.^34^ This may be related, at least in part, to the low number of episodes of severe hypoglycaemia in both age groups (15 events in 11/413 younger individuals and 12 events in 11/240 older individuals) (Table 2). Baseline characteristics that could have influenced the risk of hypoglycaemia were not markedly different between younger and older individuals, with the exception of an almost four‐fold greater prevalence of chronic moderate renal failure in older individuals (40.7% vs 10.9%) (Table 1).

Multiple comorbidities, polypharmacy and increased use of concomitant medications in older individuals with T2D may increase the risk of hypoglycaemia, and insulin metabolism may alter with age.^35^ In particular, a greater prevalence and severity of renal insufficiency in older individuals and a greater frequency of visual and/or cognitive impairment may interfere with routine self‐care in individuals with diabetes.^22^, ^36^, ^37^ Frailty^37^ can compound the burden of self‐management in older individuals and increase hypoglycaemia risk. Living alone may also increase vulnerability, as external assistance to treat severe hypoglycaemia is difficult or impossible to engage. It may also contribute, in part, to failure to identify hypoglycaemic events, and may lead to an underestimation of severe events in older individuals with T2D. The morbidity associated with hypoglycaemia may be more common and severe in older individuals. This includes a greater risk of falls and injuries such as fractures, which occur more frequently with advancing age.^36^

An important limitation of the present study is that the analysis of data from patients subdivided into age groups was not prespecified and was thus post hoc. Furthermore, information that would allow formal examination of the level of frailty in older individuals was not collected. It would have been valuable to assess whether frail patients were at higher risk of the differing severities of hypoglycaemia. In this study, a larger percentage (57.8%) of individuals in the older age group were male; this may affect the generalizability of results to the older adult population, which tends to have a greater proportion of females.

Significant strengths of the SWITCH 2 trial^25^ include the double‐blinded, treat‐to‐target design. A crossover design allowed participants to serve as their own controls when comparing treatment efficacy. In terms of assessing safety, the studies were powered with hypoglycaemia as the primary endpoint, as opposed to HbA1c, which was used in other trials that were being conducted for regulatory purposes. Furthermore, severe as well as BG‐confirmed symptomatic hypoglycaemia events were included, and all severe episodes were confirmed by adjudication. Notwithstanding the high proportion of older males in the trial, both the inclusion criteria for hypoglycaemia risk and the inclusion of older individuals in this study provide valuable insight into a population seen in real‐world practice.

To conclude, in patients with T2D, older and younger patients were at similar risk of overall symptomatic hypoglycaemia or nocturnal symptomatic hypoglycaemia, but older patients showed a tendency toward higher risk of severe hypoglycaemia. Treatment with degludec led to similar reductions in HbA1c and a similar adverse‐event profile, with a lower risk of hypoglycaemia than treatment with glargine U100, both in older and younger individuals with T2D.

## CONFLICT OF INTEREST

S. R. H. has participated in advisory panels for Eli Lilly and Company, Novo Nordisk A/S, Takeda, Sanofi‐Aventis and Boehringer Ingelheim GmbH; has been a consultant to Eli Lilly and Company and Novo Nordisk A/S; and has participated in speakers' bureaus for Novo Nordisk A/S, Eli Lilly and Company, Merck Sharp & Dohme Corp., Takeda, AstraZeneca and Boehringer Ingelheim GmbH.

J. H. D. has received research support from Dexcom, Inc., Medtronic, Novo Nordisk A/S and Senseonics; has participated in advisory panels for Novo Nordisk A/S, Sanofi and Zealand Pharma A/S; and has participated in speakers' bureaus for Novo Nordisk A/S, Roche Diabetes Care Health and Digital Solutions and Senseonics.

C. H. W. has participated in advisory panels for Abbott, AstraZeneca, Boehringer Ingelheim Pharmaceuticals, Inc., Eli Lilly and Company, Janssen Pharmaceuticals Inc. and Sanofi; has been a consultant to AstraZeneca, Janssen Pharmaceuticals Inc., Novo Nordisk Inc. and Sanofi; and has participated in speakers' bureaus for AstraZeneca, Boehringer Ingelheim Pharmaceuticals Inc., Eli Lilly and Company, Janssen Pharmaceuticals Inc., Insulet Corporation, Novo Nordisk Inc. and Sanofi.

C. T. H. and M. V. H. are employees of and hold stock in Novo Nordisk.

BMF has participated in advisory panels for Novo Nordisk A/S and Eli Lilly and Company; has been a consultant to Locemia Solutions and Zucara Therapeutics; and has participated in speakers' bureaus for Novo Nordisk A/S, Eli Lilly and Company, Roche Pharma and AstraZeneca.

## AUTHOR CONTRIBUTIONS

S. R. H., J. H. D., C. W., C. T. H., M. V. H. and B. M. F. contributed to the study design. S. R. H., J. H. D., C. W. and C. T. H. contributed to study conduct and data collection. S. R. H., J. H. D., C. W., C. T. H., M. V. H. and B. M. F. contributed to analysis of the data. S. R. H., J. H. D., C. W., C. T. H., M. V. H. and B. M. F. wrote the manuscript. Novo Nordisk contributed to the study design, statistical analyses, data interpretation, manuscript preparation and the decision to submit this manuscript for publication. All of the authors had access to study data and took full responsibility for the content of the manuscript and the decision to submit it for publication.

## Supporting information


**Table S1.** Adverse events, by system organ class and preferred term, reported ≥5% in any arm for younger (≤65 years) and older (>65 years) people, by treatment group.
**Figure S1.** Mean basal insulin dose over time, for younger (≤65 years) and older (>65 years) people.
**Figure S2.** Mean HbA_1c_ over time for younger (≤65 years) and older (>65 years) people, by treatment group.
**Figure S3.** Mean FPG over time, for younger (≤65 years) and older (>65 years) people, by treatment group.
