# Supplementary material for: Lower rates of hypoglycaemia in older individuals with type 2 diabetes using insulin degludec versus insulin glargine U100: Results from SWITCH 2
Source: Diabetes Obes Metab. 2019 Apr 15;21(7):1634–41. doi: 10.1111/dom.13708 (PMC6618254; doi:10.1111/dom.13708)
Supplement: Supplementary file 1 — Table S1. Adverse events, by system organ class and preferred term, reported ≥5% in any arm for younger (≤65 years) and older (>65 years) people, by treatment group. Figure S1. Mean basal insulin dose over time, for younger (≤65 years) and older (>65 years) people. Figure S2. Mean HbA1c over time for younger (≤65 years) and older (>65 years) people, by treatment group. Figure S3. Mean FPG over time, for younger (≤65 years) and older (>65 years) people, by treatment group. [file DOM-21-1634-s001.docx]

**Lower rates of hypoglycaemia in older people with type 2 diabetes when using insulin degludec versus insulin glargine U100: results from SWITCH 2**

Simon R Heller, J Hans DeVries, Carol Wysham, Charlotte T Hansen, Melissa V Hansen, Brian M Frier

**Supplementary appendix**

**Supplementary Table 1.** Adverse events, by system organ class and preferred term, reported ≥5% in any arm for younger (≤65 years) and older (>65 years) people, by treatment group

|  | **Insulin degludec** | | | | **Insulin glargine U100** | | | | **Total** | | | |
| --- | --- | --- | --- | --- | --- | --- | --- | --- | --- | --- | --- | --- |
|  | **N** | **%** | **E** | **R** | **N** | **%** | **E** | **R** | **N** | **%** | **E** | **R** |
| **≤65 years** |  |  |  |  |  |  |  |  |  |  |  |  |
| Infections and infestations | | | | | | | | | | | |  |
| Nasopharyngitis | 35 | 8.4 | 41 | 16.8 | 28 | 6.6 | 32 | 13.2 | 53 | 11.9 | 73 | 15.0 |
| Upper respiratory tract infection | 28 | 6.7 | 31 | 12.7 | 22 | 5.2 | 25 | 10.3 | 45 | 10.1 | 56 | 11.5 |
| **>65 years** |  |  |  |  |  |  |  |  |  |  |  |  |
| Infections and infestations | | | | | | | | | | | | |
| Bronchitis | 13 | 5.1 | 13 | 9.0 | 17 | 7.0 | 17 | 12.0 | 27 | 10 | 30 | 10.5 |
| Nasopharyngitis | 15 | 5.9 | 18 | 12.4 | 13 | 5.3 | 17 | 12.0 | 23 | 8.6 | 35 | 12.2 |
| Upper respiratory tract infection | 16 | 6.3 | 22 | 15.2 | 15 | 6.2 | 19 | 13.4 | 26 | 9.7 | 41 | 14.3 |
| Gastrointestinal disorders | | | | | | | | | | | | |
| Diarrhoea | 10 | 3.9 | 11 | 7.6 | 14 | 5.8 | 14 | 9.9 | 21 | 7.8 | 25 | 8.7 |
|  |  |  |  |  |  |  |  |  |  |  |  |  |

Safety analysis set.

%, percentage of subjects; E, number of adverse events; N, number of subjects; R, rate (number of events divided by patient-years of exposure).

**Supplementary Figure 1.** Mean basal insulin dose over time, for younger (≤65 years) and older (>65 years) people


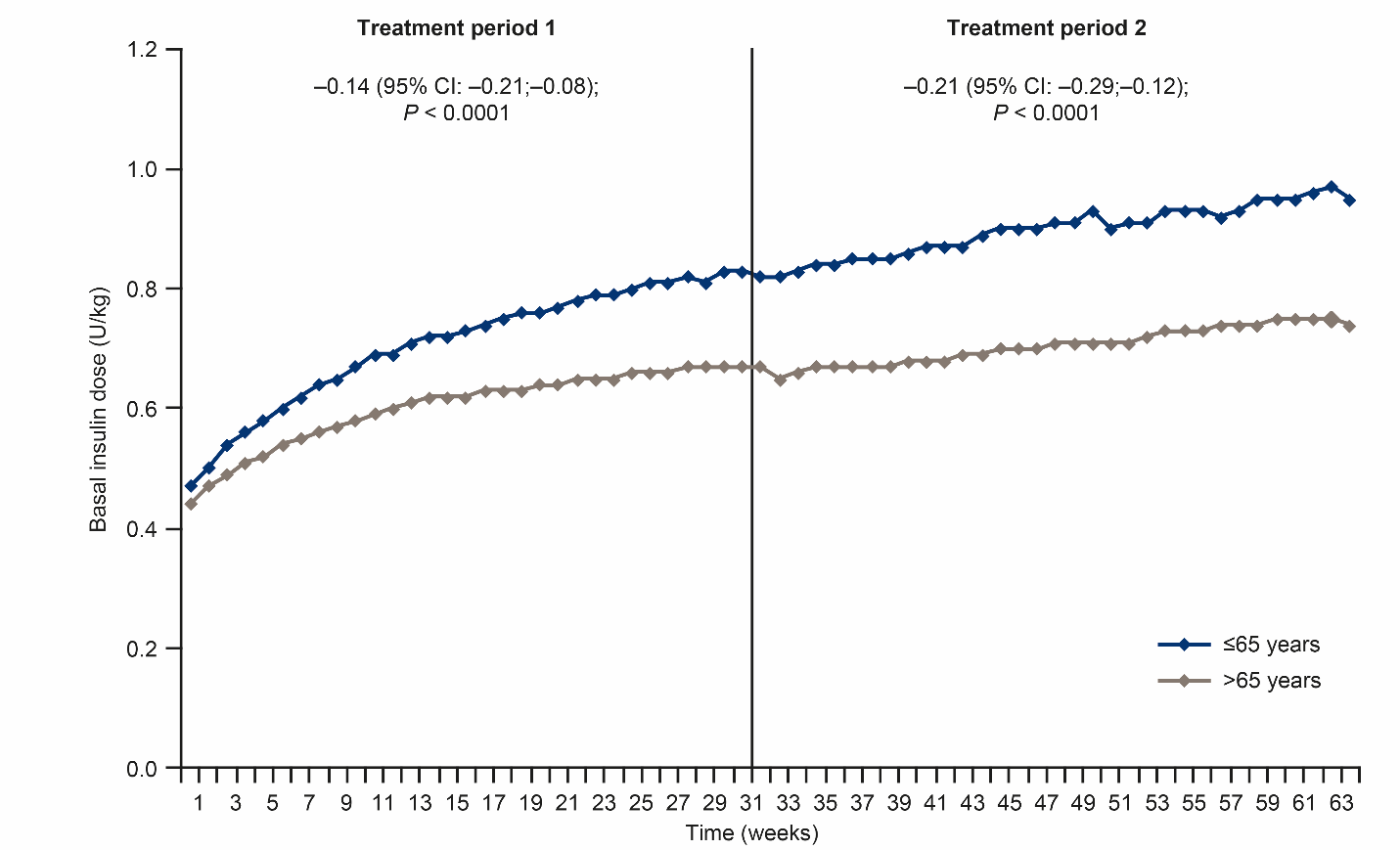
Values are age group differences, older–younger.

CI, confidence interval.

**Supplementary Figure 2.** Mean HbA_1c_ over time for younger (≤65 years) and older (>65 years) people, by treatment group

**
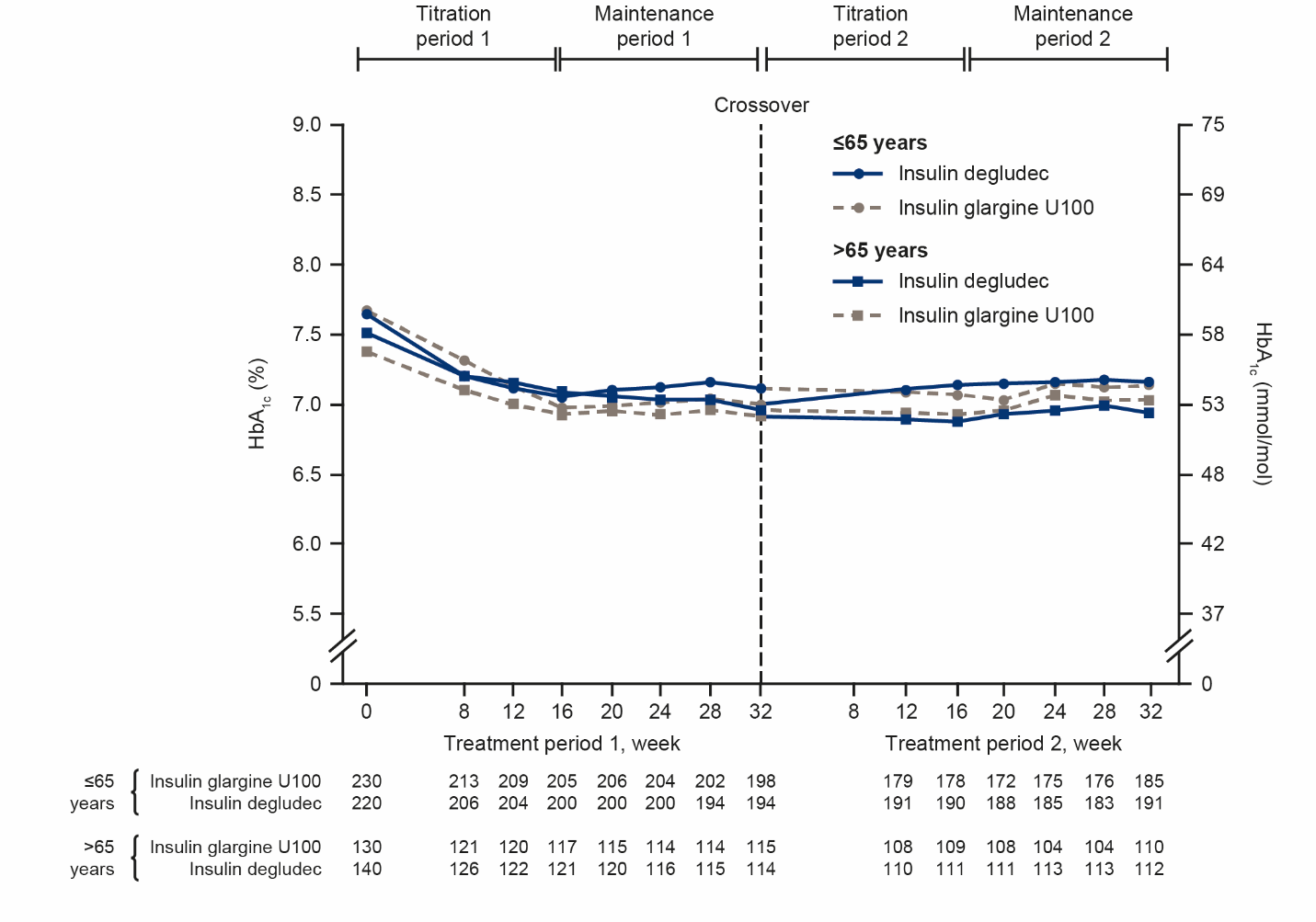
**

**Supplementary Figure 3.** Mean FPG over time, for younger (≤65 years) and older (>65 years) people, by treatment group

^
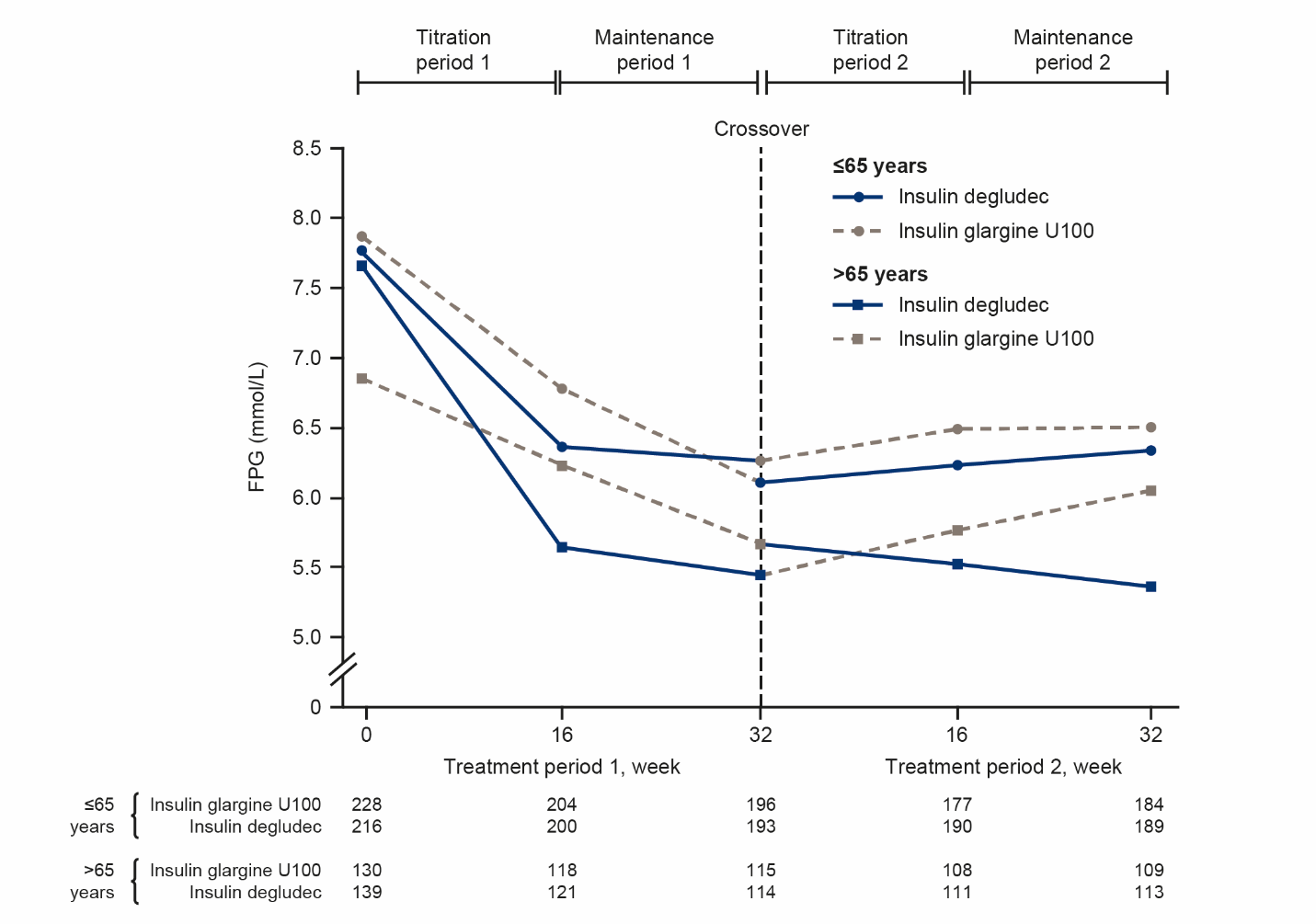
^

FPG, fasting plasma glucose.
